# Various pathways of zoledronic acid against osteoclasts and bone cancer metastasis: a brief review

**DOI:** 10.1186/s12885-020-07568-9

**Published:** 2020-11-03

**Authors:** Lianwei Wang, Dengyang Fang, Jinming Xu, Runlan Luo

**Affiliations:** 1 Department of General Surgery, Fuling Central Hospital of Chongqing City, Chongqing, China; 2 Department of Ultrasound, Fuling Central Hospital of Chongqing City, Chongqing, 408300 China

**Keywords:** Zoledronic acid, Anticancer effect, Osteoclast, Bone cancer metastasis, Anti-resorptive drug

## Abstract

Zoledronic acid (ZA) is one of the most important and effective class of anti-resorptive drug available among bisphosphonate (BP), which could effectively reduce the risk of skeletal-related events, and lead to a treatment paradigm for patients with skeletal involvement from advanced cancers. However, the exact molecular mechanisms of its anticancer effects have only recently been identified. In this review, we elaborate the detail mechanisms of ZA through inhibiting osteoclasts and cancer cells, which include the inhibition of differentiation of osteoclasts via suppressing receptor activator of nuclear factor κB ligand (RANKL)/receptor activator of nuclear factor κB (RANK) pathway, non-canonical Wnt/Ca2+/calmodulin dependent protein kinase II (CaMKII) pathway, and preventing of macrophage differentiation into osteoclasts, in addition, induction of apoptosis of osteoclasts through inhibiting farnesyl pyrophosphate synthase (FPPS)-mediated mevalonate pathway, and activation of reactive oxygen species (ROS)-induced pathway. Furthermore, ZA also inhibits cancer cells proliferation, viability, motility, invasion and angiogenesis; induces cancer cell apoptosis; reverts chemoresistance and stimulates immune response; and acts in synergy with other anti-cancer drugs. In addition, some new ways for delivering ZA against cancer is introduced. We hope this review will provide more information in support of future studies of ZA in the treatment of cancers and bone cancer metastasis.

## Background

Osteoclasts, which are abundant in the bone tissue, are multinuclear cells derived from myeloid lineage [1, 2]. Osteoclasts are known to initiate physiologic bone remodeling during bone growth, tooth eruption and fracture healing, and also are able to mediate bone loss in pathologic conditions, such as bone cancer metastasis [3, 4]. Therefore, inhibition of osteoclasts is a potential target for the treatment of bone cancer metastasis.

According to the Global Cancer Statistics 2018, there would have 18.1 million new cancer cases and 9.6 million deaths from cancer worldwide in 2018 [5]. Increasing global demographic trends and epidemiologic transitions indicate an ever-increasing cancer burden over the coming decades, particularly in low- and middle-income countries, with over 20 million new cancer cases expected annually as early as 2025 [6]. The bone is the third most common site of metastasis for a wide range of solid tumors including lung, breast, prostate, colorectal, thyroid, gynecologic, and melanoma, with 70% of metastatic prostate and breast cancer patients harboring bone metastasis [7], because of the close interactions between cancer cells and the bone marrow microenvironment which facilitates the growth of the tumors cells in the bone by providing niche, nutrients and oxygen [8]. However, the mechanism of bone cancer metastasis is very complex, including various cytokines, growth factors and other molecules involved, leading to activation of different pathways of bone resorption [9].

Zoledronic acid (ZA, C5H10N2O7P2), also called zoledronate, is the third generation of bisphosphonate (BP) with a history of only 25 years, belonging to nitrogen-containing bisphosphonate (N-BP). BP is a kind of anti-resorptive drug, and has been used clinically for near 50 years [10], which is stable pyrophosphate analogues, where a carbon atom replaces the central oxygen atom, making the P-C-P backbone non-hydrolysable [11]. Furthermore, the P-C-P backbone structure allows the BP binding to hydroxyapatite in bone tissue through the chelation of Ca^2+^ [12, 13], this is the reason why BP has high affinity with bone. Once internalized by bone-resorbing osteoclasts [14], BP affects multiple pathways to lead to effective anti-resorptive activity and induces cell apoptosis [15, 16]. ZA is the most widely used BP for its potent anti-resorptive activity, in addition, it inhibits the differentiation and apoptosis of osteoclasts [17–19]. It also has anticancer effects [15, 20], including suppressing metastasis of cancer [21, 22], inhibiting the angiogenesis [23], and the synergistic effect with other anticancer drugs [17, 20, 24]. Here, we want to elaborate the mechanisms of ZA in inhibition of differentiation and apoptosis of osteoclasts, as well as its anticancer effects, which may provide a new strategy for the treatment of cancer, especially cancer with bone metastasis.

## Inhibition of differentiation of osteoclasts by ZA

An increasing body of evidence suggests that ZA inhibits the differentiation of osteoclasts in vitro through various pathways, including inhibition of receptor activator of nuclear factor κB ligand (RANKL)/receptor activator of nuclear factor κB (RANK) pathway, non-canonical Wnt/Ca^2+^/calmodulin dependent protein kinase II (CaMKII) pathway, and prevention of macrophage differentiation into osteoclasts [19, 25, 26].

### Inhibition of RANKL/RANK pathway

Osteoclasts could be regulated by RANKL, a tumor necrosis factor (TNF)-super family cytokine produced by osteocytes and stromal cells in bone tissues [27], through binding to its receptor RANK expressed on mature osteoclasts and their precursors [28]. The dysregulation of the physiological equilibrium in the RANK/RANKL pathway also leads to the pathological remodeling associated with cancer and to the development of bone metastasis [29–31].

During osteoclast formation, the RANKL is thought to bind with RANK in osteoclast precursors, and their complex recruits TNF receptor-associated factors (TRAFs), especially TRAF6, a sensitive marker of the activity of osteoclasts, which is expressed by mature osteoclasts [32]. It activates phosphatidylinositol 3-kinase (PI3K)/protein kinase B (Akt)/mTOR pathway and subsequently nuclear factor kappa B (NF-κB) pathway [33], by promoting the phosphorylation of inhibitor of kappa Bα (IκBα) and its subsequent degradation [27], followed by increased translocation and phosphorylation of downstream p65 [34]. In addition, the RANK-TRAFs complex also activates other downstream signaling cascades, including nuclear factor of activation of T cells-1 (NFATc1) and c-fos, as well as multiple osteoclastogenesis-related genes, such as tartrate resistant acid phosphatase (TRAP), matrix metalloproteinases (MMP)-9 [35]. Moreover, mitogen-activated protein kinases (MAPKs), including C-Jun N-terminal kinase (JNK), extracellular signal-regulated kinase (Erk) and p38MAPK [17]. Downregulation of Erk inhibits the merging of osteoclast precursors, suppression of JNK impedes RANKL-stimulated the differentiation of osteoclasts [36], and the activation of p38MAPK contributes greatly to the early maturation of osteoclasts [37].

**Fig. 1:**
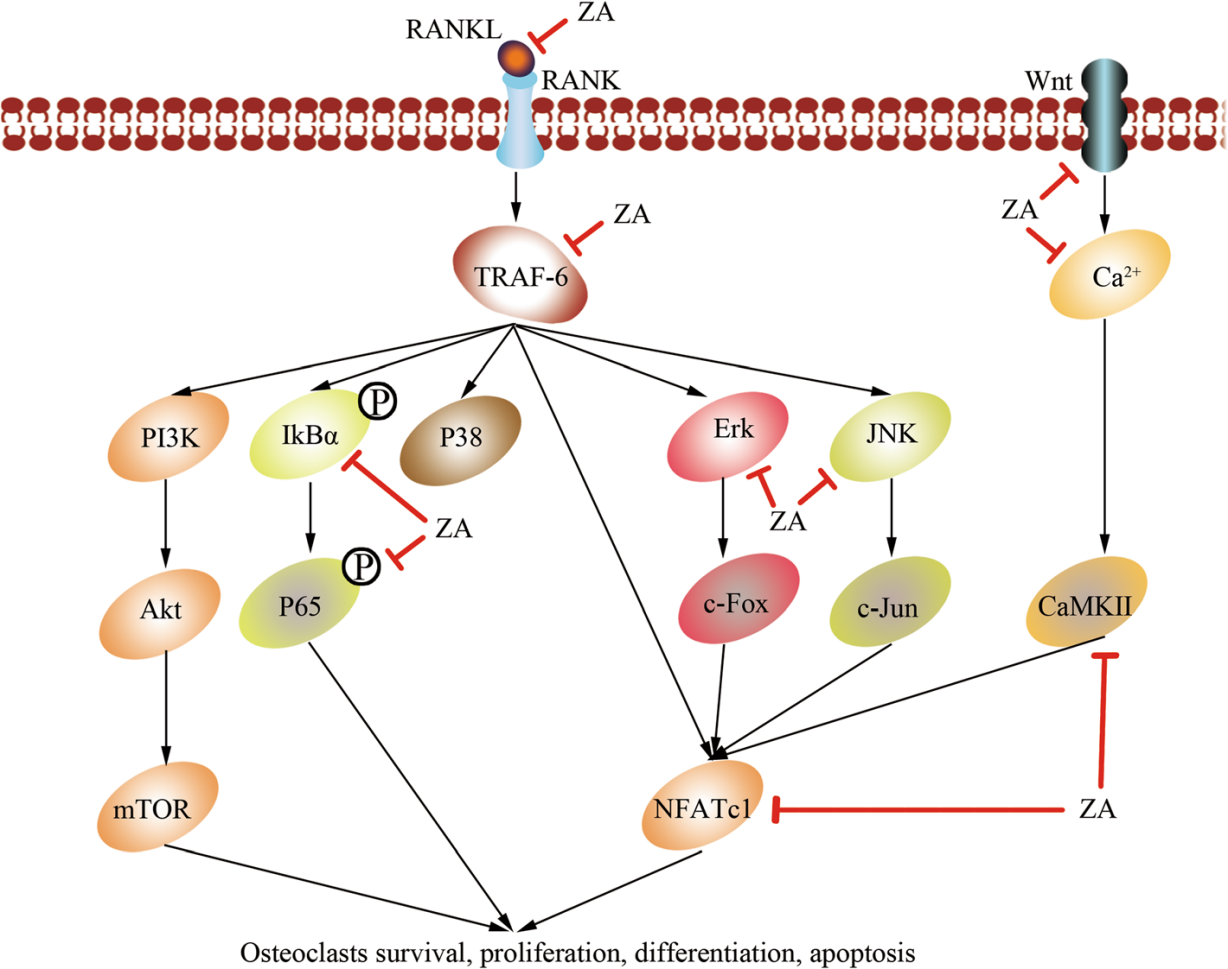
ZA inhibits the RANKL/RANK pathway. The RANKL is thought to bind with RANK, and their complex recruits TNF receptor-associated factors (TRAF6), activates phosphatidylinositol 3-kinase (PI3K)/protein kinase B (Akt)/mTOR pathway and subsequently nuclear factor kappa B (NF-κB) pathway, as well as nuclear factor of activation of T cells-1 (NFATc1), JNK, Erk and p38MAPK. ZA decreases the expression of RANKL to inhibit osteoclastogenesis, in addition, it is reported that ZA also inhibits NFATc1, suppresses NF-κB pathway, as well as inhibiting the phosphorylation of the nuclear translocation of p65, besides, ZA also inhibits non-canonical Wnt signaling through decreasing the signaling protein levels of Wnt5a, CaMKII, and the Ca2+ levels, and finally inhibits osteoclasts survival, proliferation, differentiation and apoptosis

Many experiments have demonstrated that ZA inhibits bone destruction caused by enhanced differentiation and function of osteoclasts by interrupting RANKL/RANK pathway [17, 18, 38] (Fig. 1). ZA decreases the expression of RANK to inhibit the differentiation of osteoclasts through suppressing TNF-α and RANKL [39–41]. In addition, it is reported that ZA also inhibits NFATc1 and c-fos [17, 26, 42], suppresses NF-κB pathway through promoting the deubiquitination of TRAF6 [18], as well as the phosphorylation of tyrosine and the nuclear translocation of p65 [43].

Another receptor for RANKL has to mention is leucine-rich repeat-containing G-protein-coupled receptor 4 (LGR4, also called GPR48), which competes with RANK to bind RANKL, plays a key negative feedback mechanism in the RANK-RANKL signaling pathway that negatively regulates osteoclast differentiation and bone resorption by suppressing RANK-TRAF6 signaling and activating Gαq Ca2+ induced inhibition of NFATc1 during osteoclastogenesis [44, 45]. However, whether the relationships between ZA and LGR4 has not been found until now, and this may be a new insight of ZA against osteoclastogenesis.

### Inhibition of non-canonical Wnt pathway

It is reported that non-canonical Wnt signaling, which is mainly through Ca^2+^/ CaMKII pathway, mediates osteoclastic differentiation [39, 46, 47], and the decreased non-canonical Wnt signaling results in decreases of differentiation of osteoclasts and bone resorption [48, 49]. And once osteoclasts stimulated, their Ca^2+^ levels are upregulated, and then activates calmodulin combines with CaMKII to regulate the expression of NFATc1 and TRAP, and terminally induce osteoclastic differentiation [46, 47]. ZA inhibits non-canonical Wnt signaling through decreasing the signaling protein levels of Wnt5a and CaMKII [39]. In addition, ZA significantly decreases the Ca^2+^ levels, inhibits the expression of calmodulin, CaMKII [50], and its combination, finally inhibits its differentiation [39].

### Prevention of macrophage differentiation into osteoclasts

As is known, Osteoclast is derived from myeloid lineage, including macrophage, monocyte, and osteoclast precursor cell [1, 2]. In addition to RANKL in these process, macrophage colony-stimulating factor (M-CSF) is another important cytokine, produced by mesenchymal cells in the bone marrow environment. M-CSF promotes survival and proliferation of osteoclast precursors, and their differentiation into mature phagocytes, including osteoclasts [51, 52]. ZA inhibits the activity, aggregation and migration of osteoclast precursor cells and macrophage [17, 20, 43, 53] to prevent the differentiation of osteoclasts and induce apoptosis [54]. It is demonstrated that in the presence of M-CSF, ZA also inhibits RANKL-induced upregulation of RANK mRNA to suppress the differentiation of osteoclasts [41]. Nevertheless, there is not enough evidence of the relationship between ZA and M-CSF at present, which may be another potential pathway of ZA against osteoclastogenesis.

## Induction of apoptosis of osteoclasts

Apoptosis literally means “falling away” in Greek, and occurs normally in multicellular organisms. It is a process to eliminate abnormal, damaged, or mutated cells, and plays important roles in embryonic development and adult tissue equilibrium by adjusting the physiological processes involved [55]. In humans, many cells are turned over and replaced each day through apoptosis. This process maintains a balance between the death and survival of cells and tissues [56]. It is demonstrated that ZA induces the apoptosis of osteoclasts through inhibition of the farnesyl pyrophosphate synthase (FPPS)-mediated mevalonate pathway, and induction of reactive oxygen species (ROS)-mediated apoptosis [24, 39, 57, 58].

### Inhibition of the FPPS-mediated mevalonate pathway

**Fig. 2:**
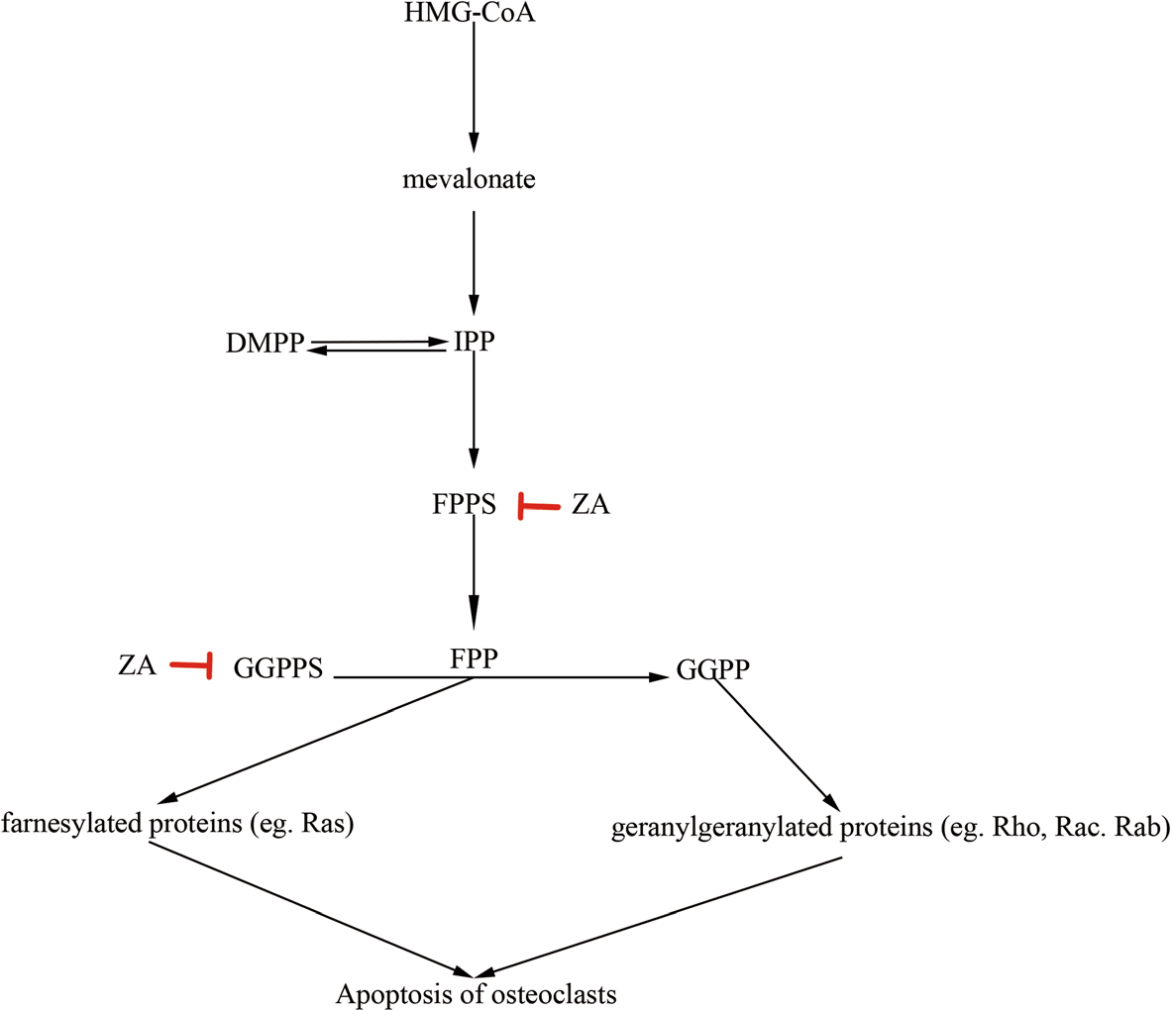
ZA inhibits mevalonate pathway. ZA inhibits farnesyl pyrophosphate synthase (FPPS) and geranylgeranyl pyrophosphate synthase (GGPPS), prevents the biosynthesis of FPP and GGPP that are required for the post-translational prenylation of small GTP-binding proteins such as Rab, Rho and Rac, leading to apoptosis of the osteoclasts

The mevalonate pathway is an important biochemical pathway in the production of cholesterol and isoprenoids, which are essential for maintaining cell membrane integrity, producing steroids and regulating cellular respiration [59]. And isoprene precursors are crucial for the prenylation of regulatory proteins involved in the control of cell proliferation, tumor progression and cell death [59]. Therefore, inhibition of the mevalonate pathway may have an impact on cellular activities that goes beyond inhibition of bone resorption [60]. It has been revealed that ZA inhibits mevalonate pathway (Fig. 2) through inhibition of FPPS, a major regulatory enzyme, to inhibit bone resorption and induce osteoclastic apoptosis [61]. FPPS, an active dimer composed of 10 α-helices with highly conserved sequences, is essential for the differentiation of osteoclast [62] through catalyzing the biosynthesis of geranyl pyrophosphate (GPP) and farnesyl pyrophosphate (FPP) [63]. FPP is the substrate of the geranylgeranyl pyrophosphate synthase (GGPPS) and it is converted into geranylgeranyl pyrophosphate (GGPP). The isoprene moieties from FPP and GGPP are post-translationally incorporated into several proteins, including many members of the Ras and Rho family of small GTPases, which control cell growth, proliferation, apoptosis and migration [11, 64, 65]. ZA inhibits FPPS and/or GGPPS, prevents the biosynthesis of FPP and GGPP that are required for the post-translational prenylation of small GTP-binding proteins such as Rab, Rho and Rac [66], leading to apoptosis of osteoclasts [38, 53, 54, 60].

### Induction of ROS-mediated apoptosis in osteoclast precursors and mature osteoclast like cells

ROS which are deleterious at high concentrations, including superoxide anion (O2-), hydrogen peroxide [67], and also nitric oxide, cause oxidative stress in the inflammatory and apoptotic process [68]. And osteoclasts are very sensitive to oxidative stress [69]. The exposure of osteoclasts to elevate oxidative stress results in cytotoxic effects due to the increased oxidative damage of DNA, proteins, and lipids, finally leads to apoptosis via the caspase-dependent pathway [70]. Recent studies have found that ZA induces apoptosis in osteoclast precursors and mature osteoclast-like cells by increasing of NADPH oxidase subunits (p91^phox^, p22^phox^, p47^phox^, and p67^phox^)/ROS to cause PI3K/AKT inactivation, glycogen synthase kinase (GSK)-3β activation, and the anti-apoptotic protein myeloid cell leukemia 1 (Mcl-1) downregulation [71]. In addition, the increased ROS also activates JNK to induce apoptosis [72]. Moreover, the expression of pro-apoptotic protein Bax is also increased by the decreased Mcl-1, which finally leads to apoptosis through sequentially activating caspase-3 dependent apoptotic pathway [71].

## Anticancer effects of ZA

ZA has a direct effect on cancer cell through inhibiting proliferation and migration of cancer cells and induces apoptosis in vitro [73] and in vivo [74] in multiple cancer types, such as neuroblastoma [21], breast cancer cells [75], prostate cancer [20, 76, 77], epidermoid cancer cells [78], pancreatic cancer [79]. Previous studies have shown that the use of ZA may significantly enhance apoptosis by elevating ROS levels in prostate carcinoma and salivary adenoid cystic carcinoma cell models [80, 81]. In addition, it is reported that ZA induces cancer cells apoptosis by inhibiting the production of RANKL in leukemia [82]. Moreover, ZA also increases the expression of pro-apoptotic protein Bax and decreases the expression of anti-apoptotic protein Bcl-2, increases the permeability of cell membrane, and induces caspase-3 dependent pathway, and finally induce apoptosis [43]. Furthermore, ZA also inhibits bone cancer metastasis through suppressing osteoclasts [21, 22, 41, 58, 83–85].

### Inducing apoptosis of cancer by ZA

The protein Ras, one of the isoprenylation of small GTP binding protein we have mentioned above, is associated with the survival pathway in cancer cells of acitivating MAPK [86], and then the Erk1/2 [86] that mediates strong anti-apoptotic effects [87]. In addition, Ras also activates the PI3K/Akt pathway to induce survival [88], moreover, Akt is activated concomitantly or independently through Ras/Raf/Mek/Erk1/2 signaling by growth factors [89, 90], and then upregulates Bcl-related proteins such as Bad and Mcl-1 to protect from apoptosis [90].

**Fig. 3:**
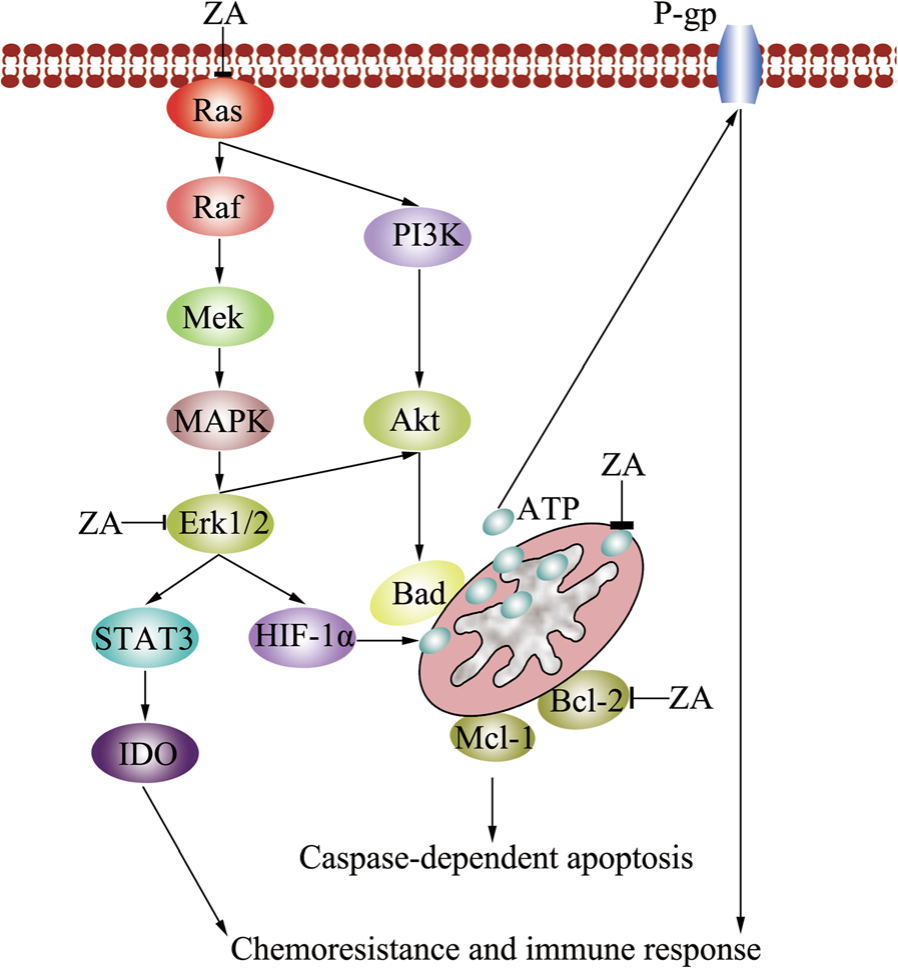
ZA induces caspase-dependent apoptosis, reverts chemoresistance and stimulats immune response in cancer cells through Ras/Erk1/2 pathway. ZA inhibits Ras, blocks the Ras-dependent Erk 1/2 and Akt pathways, and then reduces the phosphorylation of both Bcl-2 and Bad, activates the caspase-dependent apoptosis pathway to kill cancer cells. In addition, ZA also reduces the activity of hypoxia inducible factor-1 alpha (HIF-1α) through interrupting Ras/Erk1/2, and then suppresses the production of ATP, and decreases the drug efflux transporter P-glycoprotein (P-gp) to induce immunogenic cell death and reverses the tumor-induced immunosuppression. In addition, ZA also inhibits the signal transducer and activator of transcription-3 (STAT3)/ indoleamine 2,3 dioxygenase (IDO) axis via Ras/Erk1/2 pathway

It is reported that ZA inhibits Ras, blocks the Ras-dependent Erk 1/2 and Akt pathways, and then reduces the phosphorylation of both Bcl-2 and Bad, activates the caspase-dependent apoptosis pathway to kill cancer cells [76, 78, 79, 91] (Fig. 3). Interestingly, ZA is reportedly to induce apoptosis through activating caspase-3 pathway on epidermoid cancer cells [78] and breast cancer cells [92]. However, Tassone et al. found caspase-9 is activated by ZA to induce apoptosis in treatment of pancreatic cancer cells, instead of caspase-3 [79]. This may be related with tissue-specific executioners of apoptosis in different cancer types, and may have the advantage of enhancing selectivity in therapeutical intervention. In addition, ZA-mediated apoptosis is associated with cytochrome c release and consequent caspase-9 activation [79, 92]. ZA also induces actin rearrangements into cortical rings and that these events may drive the pancreatic cancer cells to the apoptotic process [79].

### Anti-angiogenesis of cancer by ZA

ZA inhibits the differentiation, migration and secretion of proangiogenic factors of mesenchymal stromal cells to suppress the growth, migration and angiogenesis of prostate cancer cells [93], including vascular endothelial growth factor (VEGF) and fibroblast growth factor-2 (FGF-2), which are related to angiogenesis, immunosuppression and migration of cancer cells [94, 95]. In addition, ZA also inhibits platelet-derived growth factor-BB (PDGF-BB), a factor released by osteoclast precursor cells could promote endothelial progenitor cells differentiating into mature endothelial cells [96], and endothelial progenitor cells to suppress angiogenesis [23].

### Anti-micrometastasis of cancer by ZA

The bone marrow microenvironment provides a site for cancer cells to escape from systemic anticancer therapy, and many bone micrometastasis are believed to be formed cancer persistence and relapse [97–99]. The growth factors and cytokines released by cancer cells enter into bone marrow microenvironment, promote osteoclasts differentiation through activating RANKL/RANK pathway with cytokines released, and finally lead to the growth and proliferation of cancer cells [100]. It is revealed that ZA significantly inhibits RANKL/RANK pathway to suppress micrometastasis of cancer. In addition, ZA also reduces the number and persistence of disseminated tumor cells in the bone marrow of patients with breast cancer [101, 102], through inhibiting chemokine C-C motif ligand 5 (CCL5)/chemokine receptor (CCR5) and IL-17B/17-BR [103]. CCL5/CCR5 regulates the coupling of cancer cells and mesenchymal stromal cells [104], and IL-17B/17-BR stimulates chemokines or enhancing inflammation [105], both of them facilitate the progression and metastasis of cancer cells. Moreover, ZA inhibits the expression of MMP-2 to suppress breast cancer metastasis [106]. In addition, it is reported that ZA suppresses the adhesion of cancer cells with extracellular matrix (ECM) to impair the process of invasion and metastasis [107]. Therefore, ZA may also be able to prevent distant metastases and local recurrence by decreasing the persistence of circulating tumor cells and disseminated cancer cells [60].

### Reverting chemoresistance and stimulating immune response by ZA

The resistance to chemotherapy and immune escape are the main causes of the failure of treatment in cancer cells. Fortunately, it has been reported that the clinically used ZA reverses chemoresistance and immunoresisitance in vitro [108, 109] (Fig. 3). ZA interrupts Ras/Erk1/2 downstream signaling pathways, and then reduces the activity of hypoxia inducible factor-1 alpha (HIF-1α), a key element in allowing cells to adapt and survive, which increases the energy metabolism and ATP synthesis in cancer cells [110], and then, suppresses the drug efflux transporter P-glycoprotein (P-gp), decreases the glycolysis and the mitochondrial respiratory chain, and finally induce a cytochrome c/caspase-dependent apoptosis in multidrug resistant cancer cells [75, 108, 109, 111]. Moreover, ZA restores the doxorubicin-induced immunogenic cell death and reverses the tumor-induced immunosuppression due to the production of kynurenine, by inhibiting the signal transducer and activator of transcription-3 (STAT3)/ indoleamine 2,3 dioxygenase (IDO) axis, which is highly activated in cancer cells. These events increased the number of dendritic cells and decreased the number of immunosuppressive T-regulatory cells infiltrating the tumors [75, 108, 109]. In addition, ZA reduces tumor burden through inhibiting FPPs, activating Vγ9 Vδ2 T cells, a special subsite of γδ T cells, to trigger activation of immunologic response through stimulating natural kill cells against cancer cells [112, 113]. ZA alters the prenylation of tumor cell and the associated macrophages to reduce tumor vascularization and prolong overall survival [114]. ZA also directly activates immunocytes of the bone marrow to kill cancer cells [115–117].

### Synergetic with other anticancer drugs

ZA combines with other anticancer drugs decreases its dosage with better effects, less cytotoxic effects and side effects. The combination of metronomic ZA and coriolus versicolor inhibits the growth breast cancer without increasing lung and liver metastasis through suppressing the expression of CD34 and MMP-2 [106]. Furthermore, it is demonstrated that the treatment of ZA with other chemotherapies in children leukemia related osteonecrosis is safe and tolerant [118]. Combination therapy with ZA and tumor-specific replicating oncolytic adenovirus DBP-301 significantly inhibits tumor-mediated osteoclast activation, tumor growth and bone destruction via suppression of Mcl-1 [24]. In addition, ZA and R115777 (Zarnestra) are synergistic in inducing both growth inhibition and apoptosis in cancer cells by disruption of Ras-dependent Erk and Akt survival pathways and consequent Bcl-related proteins-dependent apoptosis [76, 78]. Meanwhile, the bi-weekly combination of Taxotere (50 mg/m2) followed by ZA is feasible and shows promising antitumor activity through suppressing angiogenesis, tumorigenicity and metastasis in castration resistant prostate cancer patients [77].

## New ways of delivering ZA against cancer

Despite the significant antiproliferative activity of ZA on different cell lines, it has a very short plasma half-life and treads to accumulate in the bone [119]. Therefore, it is necessary to find new ways to deliver ZA in the treatment of cancer. Fortunately, it has been shown that the use of nanovectors, including liposomes (PEGylated liposomes, polysaccharides), biodegradable polymers, inorganic nanoparticles (made by metals, metal oxides or salts), hybrid nanoparticles and nanocomposite materials, can “convert” ZA in a powerful anticancer agent [120–123]. It has been reported that ZA-containing nanoparticles (N-ZA) shows superior technological characteristics in terms of mean diameter, size distribution, and ZA encapsulation efficiency, compared to ZA-encapsulating PEGylated liposomes (L-ZA). Moreover, the anti-cancer activity of N-ZA outstrips L-ZA, L-ZA outstrips free ZA in the treatment of nude mice xenografted with prostate cancer PC3 cells, both of the N-ZA and L-ZA are without any toxic effects [124]. In addition, Transferrin (Tf)-targeted N-ZA allowed the achievement of enhanced antitumor activity of ZA in a heterotopic model of glioblastoma through the acquisition of ability to cross the blood-brain barrier [125], simultaneously, it is also a novel type of easy-to-obtain nanoparticles for the delivery of ZA in the treatment of tumors [126]. All these results suggest that the future preclinical development of ZA-encapsulating nanoparticles is a trend in the treatment of human cancer.

## Conclusions

ZA is a new geminal BP that has a heterocyclic nitrogen-containing substituent. And it is the most widely used BP for its potent antiresorptive activity. Lots of experiments have demonstrated that ZA could inhibit the differentiation of osteoclasts through the inhibition of the RANKL/ RANK pathway and non-canonical Wnt/Ca2+/CaMKII pathway, prevention of macrophage differentiation into osteoclasts, induce of apoptosis of osteoclasts through inhibition of mevalonate pathway, and induction of ROS-mediated apoptosis. Moreover, ZA could also be used to treat cancer cells via the inhibition of the proliferation, viability, motility, invasion and angiogenesis of cancer cells, induction of apoptosis, and synergic with other anti-cancer drugs. As well, we also introduce the new ways for delivering ZA against cancer, and this may provide a new strategy to improve the effect of ZA in vivo. However, the side effects of ZA whether it leads to BP-induced osteonecrosis of the jaw and cancer metastasis are still in controversial. And the pharmacokinetics of ZA suggests that it is only in solution in cancer patients under two conditions: for an hour during the annual infusion and only locally in the bone microenvironment adjacent to active osteoclasts. Therefore, more focus on the side effects of ZA and the mechanisms of ZA in cancer patients will do a great favor to clinicians.

## Data Availability

Not applicable.

## Abbreviations

Akt: Protein kinase B
BP: Bisphosphonate
CaMKII: Calmodulin dependent protein kinase II
CCL5: Chemokine C-C motif ligand 5
CCR5: Chemokine receptor
ECM: Extracellular matrix
Erk: Extracellular signal-regulated kinase
FGF-2: Fibroblast growth factor-2
FPP: Farnesyl pyrophosphate
FPPS: Farnesyl pyrophosphate synthase
GPP: Geranyl pyrophosphate
GGPPS: Geranylgeranyl pyrophosphate synthase
GGPP: Geranylgeranyl pyrophosphate
GSK: Glycogen synthase kinase
HIF-1α: Hypoxia inducible factor-1 alpha
IDO: Indoleamine 2,3 dioxygenase
IκBα: kappa Bα
JNK: C-Jun N-terminal kinase
LGR4: Leucine-rich repeat-containing G-protein-coupled receptor 4
L-ZA: ZA-encapsulating PEGylated liposomes
MAPKs: Mitogen-activated protein kinases
Mcl-1: Myeloid cell leukemia 1
MMP: Matrix metalloproteinases
N-BP: Nitrogen-containing bisphosphonate
NFATc:1: Nuclear factor of activation of T cells-1
NF-κB: Nuclear factor kappa B
N-ZA: ZA-containing nanoparticles
PDGF-BB: Platelet-derived growth factor-BB
P-gp: P-Glycoprotein
PI3K: Phosphatidylinositol 3-kinase
RANK: Receptor activator of nuclear factor κB
RANKL: Receptor activator of nuclear factor κB ligand
ROS: Oxygen species
STAT3: Signal transducer and activator of transcription-3
Tf: Transferrin
TNF: Tumor necrosis factors
TRAFs: TNF receptor-associated factors
TRAP: Tartrate resistant acid phosphatase
VEGF: Vascular endothelial growth factor
ZA: Zoledronic acid
